# Evaluation of Anti-Inflammatory Effects of Six Ginsenosides and Rg1 Regulation of Macrophage Polarization and Metabolites to Alleviate Colitis

**DOI:** 10.3390/antiox14030283

**Published:** 2025-02-27

**Authors:** Qian Qu, Wenbo Zhang, Zhaoying Xuan, Rong Chen, Yimu Ma, Yiwen Huang, Yifan Hu, Yulin Lin, Mengjie Liu, Weijie Lv, Shining Guo

**Affiliations:** 1 College of Veterinary Medicine, South China Agricultural University, Guangzhou 510642, China; qianqu@scau.edu.cn (Q.Q.); 20222029021@stu.scau.edu.cn (W.Z.); xuanzhaoying@stu.scau.edu.cn (Z.X.); chenrong@stu.scau.edu.cn (R.C.); ym_ma@stu.scau.edu.cn (Y.M.); even1024@stu.scau.edu.cn (Y.H.); yifanhu@stu.scau.edu.cn (Y.H.); linyulin@stu.scau.edu.cn (Y.L.); mengjieliu@stu.scau.edu.cn (M.L.); 2 Guangdong Research Center for Veterinary Traditional Chinese Medicine and Natural Medicine Engineering Technology, Guangzhou 510642, China

**Keywords:** colitis, ginsenoside, macrophage polarization, Rg1, metabolites

## Abstract

In ginseng, several ginsenosides have been demonstrated to alleviate dextran sulfate sodium (DSS)-induced colitis, especially the six ginsenosides in this study. However, which ginsenoside has the most potent anti-inflammatory effect and may be selected as a promising candidate for the treatment of colitis remains unclear. A cell inflammation model was induced by lipopolysaccharide (LPS) for 12 h and mouse colitis was induced by sterile water containing DSS lasting seven days. Cytokines associated with inflammation, pyroptosis, and ferroptosis were assessed by quantitative real-time PCR (qPCR), the level of reactive oxygen species (ROS) and changes in macrophage polarization were tested by flow cytometry, and analysis of intestinal metabolites by LC-MS/MS was performed. The results in this study displayed that among the six ginsenosides, Rf, Rg1, and Rg3 were the most effective in reducing LPS-induced inflammation in cells. Compared with Rg3 and Rf, Rg1 was superior in restoring body weight and the length of colon, decreasing the disease activity index (DAI), and reducing splenomegaly and colon inflammation. Meanwhile, Rg1 significantly decreased the expression of M1-related pro-inflammation cytokines and increased the expression of M2-related anti-inflammation cytokines. Rg1 also decreased CD86^+^M1 macrophages and polarized them towards CD206^+^M2 macrophages. The 700 targeted gut metabolite assays revealed that Rg1 treatment brought the metabolite composition closer to that of DSS-naive mice, while six key metabolites, including dodecanoylcarnitine, isobutyric acid, and decanoylcarnitine, and so on, all were significantly reversed. Our results demonstrated that among the six ginsenosides, Rg1 had the most extraordinary anti-inflammatory effect in LPS-induced cells and DSS-induced mice, and, more importantly, it blunted colitis through regulating macrophage polarization and intestinal metabolites.

## 1. Introduction

Ginseng (Panax ginseng Meyer) is known as the king of herbs and is a perennial herbaceous plant in the family Araliaceae. Ginseng is largely grown in China, South Korea, and the United States and has been used for thousands of years. Ginseng is a traditional Chinese medicine used to tonify five zang organs, calm nerves, improve eyesight, and prolong life. It was first recorded in Shen Nong’s Herbal Classic [1]. Ginseng is an essential ingredient in many classic traditional Chinese medicine formulas, such as Ginseng Decoction, Shen Ling Bai Zhu San (SLBZS), and so on, which are used to cure gastrointestinal diseases [2,3]. Modern pharmacological studies show that ginseng has multiple functions and has the potential to deal with Alzheimer’s disease, vascular dementia, skin diseases, etc. [4,5,6]. Ginseng has also been reported as a potential therapeutic candidate for inflammatory bowel disease (IBD) in several studies, and the active ingredients, including polysaccharides and saponins, have also been shown to attenuate colitis [7,8,9].

Ginseng contains a large number of different active ingredients, among which ginsenosides are the most important class of active substances. More than 100 kinds of ginsenosides have been clearly identified in ginseng [10]. Ginsenosides have been shown to have significant anti-inflammatory effects, among which the most commonly studied ginsenosides are Rg1, Re, Rg3, Rd, Rb1, and Rh1, and updated study includes the more anti-inflammatory ginsenosides Rc, Rf, Rg5, Rg6, Ro, and so on [11]. Recent studies have shown that ginsenosides weaken inflammation by regulating cellular immunity, including T cell and macrophage proliferation and differentiation, repairing the intestinal barrier, balancing gut microbiota and metabolites, inhibiting inflammatory pathways, and activating anti-inflammatory signals [8]. SLBZS is a traditional Chinese medicine formula used to treat gastrointestinal diseases, consisting of 10 Chinese herbs, among which ginseng is the main ingredient [3]. In our previous study, SLBZS was divided into four different effective components by organic reagents. Among them, the *n*-butanol extract, which was detected to contain ginsenosides Rg1, Re, and Rb1 by HPLC, was superior to the other three extracts in reducing colitis, while the other three did not detect these three ginsenosides. In addition, the *n*-butanol extract of SLBZS also contains more ginsenosides, such as Rg3, Rd, Rf, etc., which have been shown to alleviate colitis or intestinal inflammation [3,11]. However, which type of ginsenoside plays a critical role in reducing colitis remains unclear. Therefore, in this study, the anti-inflammatory effects of these six different ginsenosides were explored together.

Inflammatory bowel disease displays chronic inflammation occurring in the intestines and is characterized by diarrhea and abdominal pain [3]. Macrophages, which are polarized phenotypically by the microenvironment to mount specific functional programs, play a critical role in inflammatory bowel disease. Based on differences in cytokine secretion and metabolism, macrophages include two types, M1 and M2 [12]. Activating M1 Macrophages will release more pro-inflammatory factors, exacerbating the development of colitis; M2 macrophages release anti-inflammatory factors to alleviate inflammation [13]. Previous studies have shown that inducing polarization of M2 macrophages is an effective method for treating IBD [14]. Huang et al. [15] and Zhao et al. [16] showed that promoting the polarization of M2 macrophages contributes to the relief of intestinal inflammation, the repair of damaged intestinal tissue, and the inhibition of ferroptosis, making it a candidate drug for the treatment of inflammatory bowel disease. Ferroptosis is a new type of programmed cell death proposed in 2012, which is closely related to iron metabolism, lipid peroxidation, and amino acid metabolism [17]. The induction of xCT/GPX4 and Nrf2 antioxidant signals are important pathways to alleviate ferroptosis, thereby further alleviating colitis [18]. Studies have shown that iron overload increases the release of M1-related cytokines and decreases markers of M2 [19]. Pyroptosis is a programmed cell death that is different from apoptosis and is accompanied by the release of a large number of pro-inflammatory factors. It has been shown that downregulation of pyroptosis signaling can ameliorate experimental colitis [20]. Therefore, balancing macrophage polarization and inhibiting ferroptosis and pyroptosis, which contribute to the alleviation of inflammation or colitis, can be the key to evaluating the efficacy of anti-inflammatory drugs.

So far, the anti-inflammatory effects of different ginsenoside monomers, which have been shown to have anti-inflammatory or anti-colitis properties, have not been consistently compared. In the current study, to evaluate the anti-inflammatory potential of several key ginsenosides, including Rg1, Rg3, Rd, Re, Rb1, and Rf, macrophage polarization, pyroptosis, and ferroptosis were examined in an LPS-induced RAW264.7 macrophage inflammation model. The in vivo DSS-induced inflammation model was then used to further investigate the anti-inflammatory effects of the three ginsenosides, which have been shown to have optimal anti-inflammatory effects in vitro.

## 2. Materials and Methods

### 2.1. Reagents and Antibodies

The ginsenosides Rg1, Rg3, Rd, Rb1, Re, and Rf, (purity ≥ 98.0%) were purchased from Chengdu Biopurify Phytochemicals Ltd. (Chengdu, China), 2′,7′-Dichlorofluorescin Diacetate (DCFH-DA) was purchased from Sigma-Aldrich Trading Co., Ltd. (Shanghai, China), and dextran sulfate sodium (36,000–50,000 Da) was purchased from MP Biotechnology Co., Ltd. (Santa Ana, CA, USA). PE/Cyanine7 anti-mouse CD86 was obtained from BD Biosciences (Franklin Lakes, NJ, USA) and FITC anti-mouse/human CD11b, PE anti-mouse F4/80, and Alexa Fluor^®^ 647 anti-mouse CD206 (MMR) were from Biolegend (San Diego, CA, USA). Methanol and acetonitrile were all LC-MS level (Thermo Fisher Scientific, Waltham, MA, USA), we also used ammonia formate (Honeywell Fluka, Charlotte, NC, USA), and formic acid (Dikma Technologies Inc., CA, USA) was the analytical reagent.

### 2.2. Cell Culture

RAW264.7 macrophage cells were grown in high glucose Dulbecco’s modified eagle medium (DMEM), supplemented with fetal bovine serum, 1% penicillin, and streptomycin (Gibco; Thermo Fisher Scientific, Inc., Waltham, MA, USA) at an incubation temperature of 37 °C in a Forma™ II series water jacket CO_2_ incubator (Thermo Fisher Scientific, Inc., Waltham, MA, USA) containing a 5% concentration of carbon dioxide.

### 2.3. Measurement of Cell Viability

The cell viability was tested by a Cell Counting Kit-8 (CCK-8) purchased from Beyotime Biotechnology Co., Ltd. (Shanghai, China). The cells were enclosed in a 96-well plate at 2 × 10^4^ cells/well with 6 replicates per treatment. When the cells were completely adherent, they were treated with different concentrations (0 μM–100 μM) of Rg1, Rg3, Rd, Re, Rb1, and Rf for 24 h. The supernatant was removed, medium containing 10% CCK8 solution from Cell Counting Kit-8 was added for 2 h, and then cell viability was analyzed on a Multiskan FC Microplate Reader (Thermo Fisher Scientific).

### 2.4. Quantitative Real-Time PCR (qPCR) for Cells

The cell plating method is the same as above, accompanied by different cell densities (2 × 10^5^ cells/well, 3 replicates per treatment), treated with 6 ginsenosides at concentrations of 25 μM (Low) or 50 μM (High). Then, LPS (1 μg/mL) was inserted for 12 h. The supernatant was deleted from the 12-well plate and the cells were extracted using Trizol reagent (Vazyme Biotech Co., Ltd, Nanjing, China). The cDNA was synthesized by HiScript III RT SuperMix (Vazyme, Nanjing, China) for qPCR (+gDNA wiper) and quantitated with the ChamQ universal SYBR qPCR Master Mix (Vazyme, Nanjing, China) according to the manufacturer’s instructions. The amplification program was as follows: predenaturation at 95 °C for 30 s, 40 cycles at 90 °C for 15 s and 60 °C for 30 s, and melting curves for 15 s. The analyses of the samples were performed using qTOWER^3^ (Analytik Jena GmbhH, Jena, Germany), and the data were calculated using the 2^−ΔΔCT^ method to calculate relative gene expression with β-Actin as the control. The primer sequences are given in Table 1. The colon tissue was treated using the same method as above.

### 2.5. Measurement of ROS

The cell plating method is the same as above, 1 × 10^5^ cells/well, and each treatment was repeated 3 times, treated with 6 ginsenosides at concentrations of 25 μM or 50 μM, respectively. Then, LPS (1 μg/mL) was inserted for 12 h. After incubation, the supernatant was removed and serum-free medium containing 20 μM DCFH-DA was added for 30 min. Then, the samples were washed with PBS three times and analyzed by CytoFLEX flow cytometry (Beckman Coulter, Brea, CA, USA).

### 2.6. Animal Experiments

Sixty male C57BL/6J mice around 7 weeks of age were divided into 5 groups of 12 each (Blank, DSS, DSS + Rf, DSS + Rg1, and DSS + Rg3) and housed in a barrier environment with temperature of 18–22 °C and humidity of 50–55%, with four per cage on a 12-h day–night cycle. The animal experimental procedures in our study have been approved by the Animal Ethics Committee of the South China Agricultural University, and the mice were fed in the animal experiment center of South China Agricultural University (approval number: SYXK 2022-0136).

The mouse colitis model was induced by continuous drinking of purified water containing DSS (2.5%) lasting seven days. Then, the mice were orally administered Rg1, Rg3, and Rf at a dose of 75 mg/kg for 7 consecutive days. The mice in blank group were given normal purified water for 7 days and orally administered purified water at the same dose as ginsenosides for 7 days. Body weight was weighed, and diarrhea and bleeding in discharged feces were evaluated every other day. At the endpoint, mice were euthanized by cervical dislocation, and colon tissue was harvested for qPCR (middle segment of the colon), H&E staining (distal segment of the colon), and flow cytometry analysis (whole segment of colon), and feces were collected for metabolite analysis.

### 2.7. Quantitative Real-Time PCR (qPCR) for Colon Tissue

The total RNA of colon tissue was extracted using Trizol reagent. Then, the cDNA synthesis and quantification were performed as described in Section 2.4.

### 2.8. Histopathology

The colon tissues were set with formaldehyde, dehydrated, embedded, sliced, and then stained with H&E staining. Histological changes were observed with a Nikon Eclipse ci light microscope and photographed with a Nikon DS-U3 imaging system (Nikon Corporation, Tokyo, Japan).

### 2.9. Enzyme-Linked Immunosorbent Assay (ELISA)

The detection of tumor necrosis factor (TNF-α), interleukin (IL)-1β, and IL-6 in serum was performed using an enzyme-linked immunosorbent assay kit according to the manufacturer’s instructions (Shagnhai Coibo Bio Technology Co., Ltd., Shanghai, China) and analyzed using a Multiskan FC microplate reader (Thermo Fisher Scientific, Inc.).

### 2.10. Flow Cytometry

Fresh colon tissue was separated and placed in pre cooled PBS containing 2% fetal bovine serum, completely washed of feces, and then incubated in a buffer containing 1 mM DTT and 2 mM EDTA at 37 °C for 20 min. After the buffer was removed, the tissue was cut into pieces and incubated in a medium containing 20 μg/mL DNase-I and 0.5 mg/mL Collagenase Ⅷ for 30 min with shaking at 200 rpm. Finally, the samples were sifted through 70 μm and stained with fluorescently conjugated antibodies (CD11b, F4/80, CD86, and CD206) in PBS containing of 2% FBS and 2 mM EDTA. The results were performed using CYTOFLEX (Beckman Coulter), and the data were analyzed by FlowJo v.10.8.

### 2.11. LC-MS/MS

The feces were mixed with sterilized water and centrifuged at 18,000 rpm to obtain the supernatant. The supernatant was added to the plate, the derivatization reagent and working solution were added in sequence, vortexed for 60 min at 40 °C, and then centrifuged at 4000 rpm for 10 min. Finally, the sample was added to the diluent and centrifuged before preparation for the machine.

The sample extracts were analyzed using Waters UPLC I-Class Plus (Waters, Milford, MA, USA) equipped with QTRAP 6500 Plus (SCIEX, Framingham, MA, USA). Chromatography Conditions: The column used is BEH C18 (2.1 mm × 10 cm, 1.7 μm, waters). The mobile phase is water containing 0.1% formic acid (Solvent A) and acetonitrile containing 30% isopropanol (Solvent B). Elution was performed using the following gradient: 0–1.00 min, 5% B; 1.00 m–5.00 min, 5% B; 5.00–9.00 min, 70% B; 9.00–11.00 min, 50% B; 11.00–13.50 min, 22% B; 13.5–14.00 min, 95% B; the flow speed above is 0.4 mL/min; 14.00−16.00 min, 100% B, the flow speed is 0.6 mL/min; 16.00–18.00 min, 5% B, the flow speed is 0.4 mL/min. Mass Spectrometry Conditions: A QTRAP 6500 Plus with ESI Turbo ion spray interface (SCIEX, Framingham, MA, USA) was equipped. Ion source parameters are as follows: ion source temperature: 400 °C; ion spray voltage: 4500 V (positive mode) and −4500 V (negative mode); ion source gas 1 (GS1), ion source gas 2 (GS2), and curtain gas were set to 60, 60, and 35 psi, respectively. The column temperature is 40 °C.

### 2.12. Statistical Analysis

Statistical analysis was performed by SPSS 26 software. Figures were created using GraphPad 8. Comparison between two groups was performed using a Student’s *t*-test or Mann–Whitney test. Otherwise, differences were compared by ANOVA with LSD or non-parametric test with Kruskal–Wallis among three or more groups. * *p* < 0.05, ** *p* < 0.01, and *** *p* < 0.001 compared with the control/blank group; # *p* < 0.05, ## *p* < 0.01, and ### *p* < 0.001 compared with the LPS/DSS group.

## 3. Results

### 3.1. Anti-Inflammatory Effect of Different Ginsenosides

**Figure 1:**
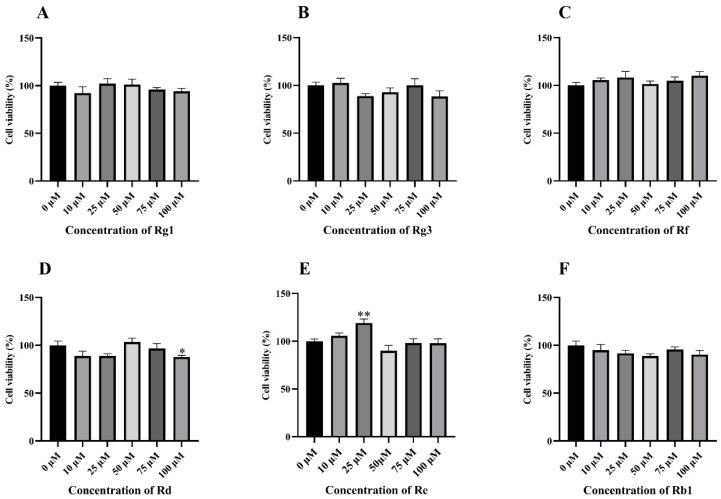
The cell viability of different ginsenosides on LPS-induced RAW264.7 cells. The cell viability of (**A**) Rg1, (**B**) Rg3, (**C**) Rf, (**D**) Rd, (**E**) Re, and (**F**) Rb1 on LPS-induced RAW264.7 cells for 12 h were determined by CCK8 (*n* = 6). The concentration of different ginsenosides were 0 μM, 10 μM, 25 μM, 50 μM, 75 μM, and 100 μM, respectively. * *p* < 0.05, ** *p* < 0.01 compared with the 0 μM group.

**Figure 2:**
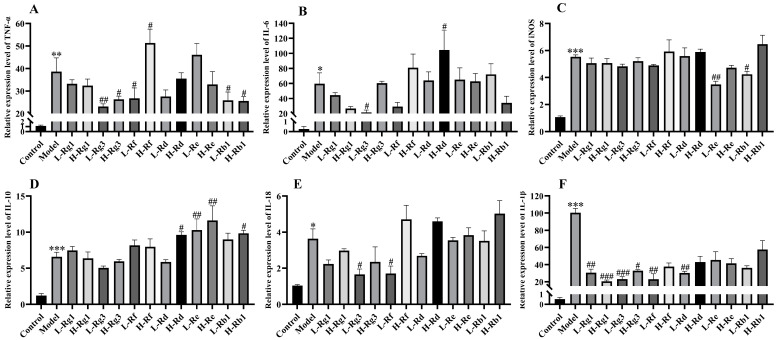
Anti-inflammatory effect of different ginsenosides on LPS-induced RAW264.7 cells. The mRNA expression levels of (**A**) TNF-α, (**B**) IL-6, (**C**) iNOS, (**D**) IL-10, (**E**) IL-18, and (**F**) IL-1β were detected by qPCR (*n* = 4). L: 25 μM, H: 50 μM. * *p* < 0.05, ** *p* < 0.01, and *** *p* < 0.001 compared with the control group; ^#^ *p* < 0.05, ^##^ *p* < 0.01, and ^###^ *p* < 0.001 compared with the model group.

To evaluate the anti-inflammatory effects of six ginsenosides, which are the most commonly studied, an LPS-induced RAW264.7 cell inflammation model was established. Firstly, the cellular activity of six commonly studied ginsenosides was observed (Figure 1). CCK8 results showed that six ginsenosides were non-toxic on RAW264.7 cells at concentrations of 25 μM and 50 μM. The level of inflammatory factor TNF-α was significantly reduced by Rg3, Rf, and Rb1 (Figure 2A), the level of IL-6 was remarkedly reduced by Rg3 (Figure 2B), the expression of iNOS was particularly reduced by Re and Rb1 (Figure 2C), the level of IL-18 was remarkedly reduced by Rg3 and Rf (Figure 2E), and the relative expression of IL-1β mRNA was particularly reduced by Rg3, Rf, Rg1, and Rd (Figure 2F). The mRNA expression of the anti-inflammatory cytokine IL-10 was obviously upregulated by Re, Rd, and Rb1 (Figure 2D). However, compared with the blank group, the LPS-induced inflammation model also upregulated the mRNA level of IL-10 (Figure 2D). However, the effects of Rg3, Rf, and Rd on inflammatory cytokines decreased with increasing doses. Pooled data showed that among the six ginsenosides, Rg3 and Rf were the most effective in decreasing inflammation.

### 3.2. The Inhibitory Activity of Different Ginsenosides on Pyroptosis

**Figure 3:**
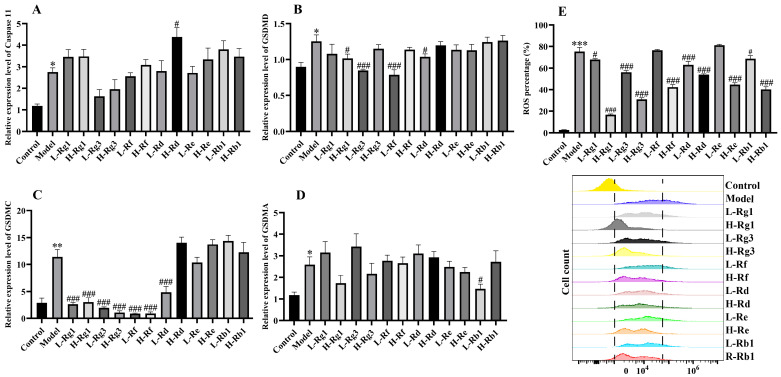
Effect of different ginsenosides on the inhibition of pyroptosis and ROS in LPS-induced RAW264.7 cells. The mRNA expression levels of (**A**) Caspase-11, (**B**) GSDMD, (**C**) GSDMC, and (**D**) GSDMA were determined by qPCR. (**E**) Changes in ROS production after different ginsenoside treatments were measured by flow cytometry (*n* = 4). L: 25 μM, H: 50 μM. * *p* < 0.05, ** *p* < 0.01, and *** *p* < 0.001 compared with the control group; ^#^ *p* < 0.05, and ^###^ *p* < 0.001 compared with the model group.

The occurrence of pyroptosis depends on the gasdermins (GSDMs) protein family and is accompanied by the release of a large number of proinflammatory factors. The effects of the six ginsenosides on IL-18 and IL-1β, a pyroptosis-related cytokine, have been analyzed above (Figure 2E,F). Although LPS induced an obvious increase in the mRNA expression of Caspase11, GSDMD, GSDMC, and GSDMA in RAW264.7 cells (Figure 3A–D), the mRNA expression of both GSDMD and GSDMC was drastically inhibited by Rg3, Rd, Rf, and Rg1 (Figure 3B,C). Among them, the inhibitory effect of Rd on GSDMC expression and the inhibitory effect of Rb1 on GSDMA did not appear to be dose-dependent. Collecting data, the results showed that among these six ginsenosides, Rg1, Rg3, Rf, and Rd were the optimum in inhibiting pyroptosis.

### 3.3. The Effect of Different Ginsenosides on ROS

Inflammation and ROS are a bidirectional regulatory process that promotes each other’s occurrence. In this study, the results indicated that LPS significantly induced cells to release ROS, while all six ginsenosides markedly inhibited ROS levels, with Rg1 being the most outstanding by flow cytometry analysis (Figure 3E). Therefore, the results of LPS-induced inflammation indicated that Rg1, Rg3, and Rf were the most prominent in inhibiting inflammation.

### 3.4. The Effect of Different Ginsenosides on Ferroptosis

**Figure 4:**
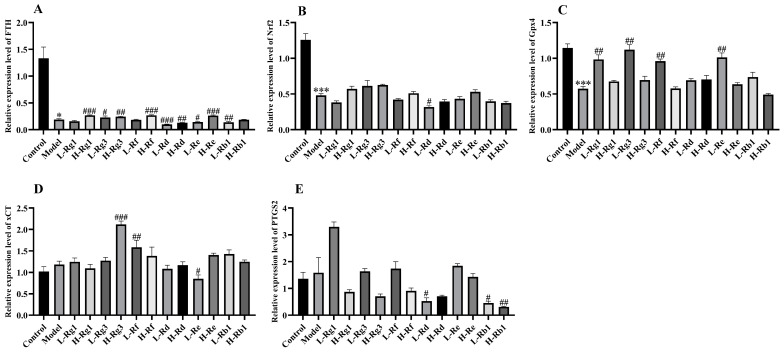
Effect of different ginsenosides on the inhibition of ferroptosis in LPS-induced RAW264.7 cells. The mRNA expression levels of (**A**) FTH, (**B**) Nrf2, (**C**) Gpx4, (**D**) xCT, and (**E**) PTGS2 were determined by qPCR (*n* = 4). L: 25 μM, H: 50 μM. * *p* < 0.05, and *** *p* < 0.001 compared with the control group; ^#^ *p* < 0.05, ^##^ *p* < 0.01, and ^###^ *p* < 0.001 compared with the model group.

Then, the effects of different ginsenosides on ferroptosis were observed, and ferroptosis is closely related to oxidative stress. The mRNA expression of ferritin heavy chain (FTH), nuclear factor erythroid 2-related factor 2 (Nrf2), and glutathione peroxidase 4 (Gpx4) was significantly reduced in the model group, but FTH and Gpx4 expression was significantly upregulated by Rg1, Rg3, Rf, and Re (Figure 4A,C), and Nrf2 expression was upregulated by Rg1 and Rg3 (Figure 4B). Although there was no significant change in the relative expression levels of xCT and prostaglandin-endoperoxide synthase 2 (PTGS2) between the blank group and the model group, the relative expression of xCT was significantly upregulated by Rg3 and Rf and significantly downregulated by Re, while the relative expression of PTGS2 was significantly downregulated by Rd and Rb1 (Figure 4D,E). However, ginsenoside treatment did not show a dose-dependent effect on Gpx4 expression.

### 3.5. The Effect of Rg1, Rg3, and Rf on Improving Colitis

**Figure 5:**
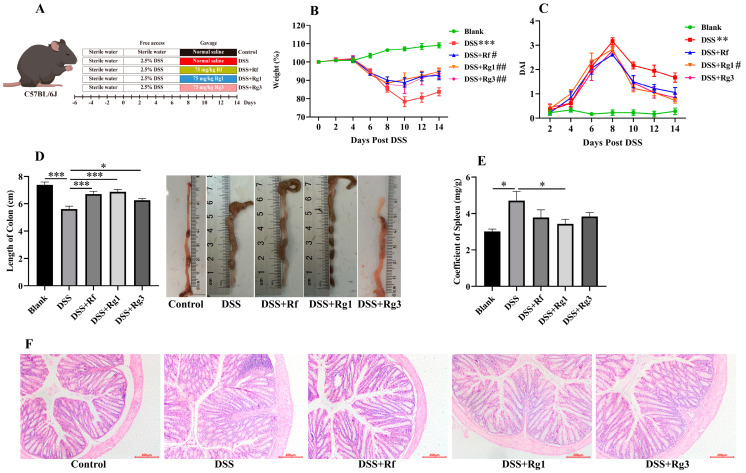
Supplementation with Rg1, Rg3, and Rf blunted DSS-induced colitis. (**A**) Schematic diagram for the establishment of colitis mice model with Rg1, Rg3, and Rf. Effect of Rg1, Rg3, and Rf on (**B**) body weight, (**C**) DAI scores, (**D**) colon lengths, and (**E**) coefficient of the spleen in DSS-induced mice (*n* = 6). (**F**) Representative pictures of H&E stained histological sections of the colon in mice. Scale bars = 200 μm. * *p* < 0.05, ** *p* < 0.01, and *** *p* < 0.001 compared with the blank group; ^#^ *p* < 0.05 and ^##^ *p* < 0.01 compared with the DSS group.

To further compare the anti-inflammatory effects of Rg1, Rg3, and Rf in vivo, the mice colitis model was induced by DSS and then treated with these three ginsenosides (Figure 5A). After Rg1 and Rg3 treatment, the body weight of DSS-induced mice was significantly restored, but it was still lower than that of the blank group (Figure 5B). DAI was considerably reduced in colitis mice after Rg1 gavage (Figure 5C). In addition, Rg1, Rg3, and Rf reduced colonic shortening, while only Rg1 reduced splenomegaly in colitis mice (Figure 5D,E). H&E staining showed that Rg1, Rg3, and Rf all alleviated colonic inflammatory cell infiltration in varying degrees (Figure 5F). Taken together, these results showed that Rg1 had the most noticeable effect in relieving colitis in mice.

### 3.6. The Effect of Rg1, Rg3, and Rf on Serum Cytokines and the Intestinal Barrier

**Figure 6:**
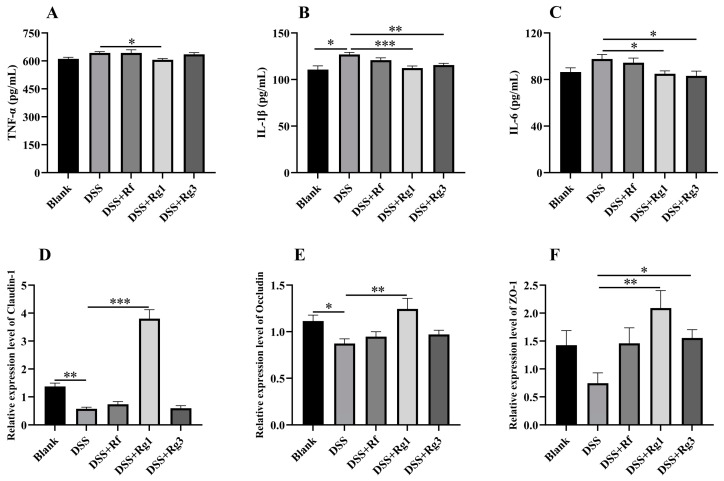
Supplementation with Rg1, Rg3, and Rf inhibited peripheral blood inflammation and repaired the intestinal barrier in DSS-induced colitis. The concentration of (**A**) TNF-α, (**B**) IL-1β, and (**C**) IL-6 were determined in serum by ELISA. The mRNA expression levels of (**D**) Claudin1, (**E**) Occludin, and (**F**) ZO-1 were measured by qPCR (*n* = 5). * *p* < 0.05, ** *p* < 0.01, and *** *p* < 0.001 compared with the blank/DSS group.

Then, the inflammatory cytokines in peripheral blood and the function of colonic intestinal barrier genes were observed (Figure 6). The concentrations of inflammatory cytokines TNF-α, IL-1β, and IL-6 in serum were increased by DSS, but only IL-1β showed significant differences. However, treatment with Rg1 significantly decreased the levels of these three cytokines, while Rg3 only significantly reduced the levels of IL-1β (Figure 6A–C). The relative mRNA expression of tight junctions Claudin1, Occludin, and ZO-1 was also significantly reduced in DSS-induced colitis mice, while Rg1 significantly upregulated their relative mRNA expression and Rg3 significantly upregulated the relative mRNA expression of ZO-1 (Figure 6D–F).

### 3.7. Modulation of Macrophage Polarization by Rg1

**Figure 7:**
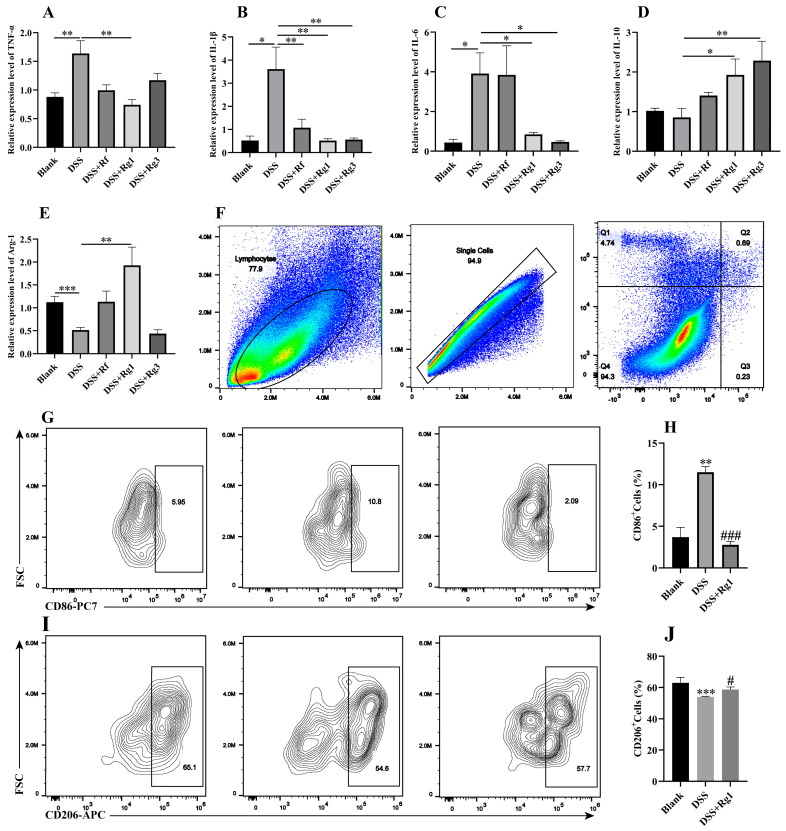
Rg1 modulated macrophage polarization in DSS-induced mice. The mRNA expression levels of (**A**) TNF-α, (**B**) IL-1β, (**C**) IL-6, (**D**) IL-10, and (**E**) Arg-1 in colon tissue were determined by qPCR. (**F**) Gating strategy to sort macrophage polarization by multicolor flow cytometry. Gating to sort (**G**) M1 macrophage by CD86 antibody and (**I**) M2 macrophage by CD206 antibody. Changes in (**H**) M1 macrophage and (**J**) M2 macrophage after Rg1 treatment in the colon of colitis by flow cytometry (*n* = 5). * *p* < 0.05, ** *p* < 0.01, and *** *p* < 0.001 compared with the blank group; ^#^ *p* < 0.05, and ^###^ *p* < 0.001 compared with the DSS group.

Colitis leads to inflammation of colon tissue and splenomegaly; therefore, we further analyzed the effects of Rg1, Rg3, and Rf on colonic and splenic inflammation. In the colon of DSS-induced mice, Rg1 significantly downregulated the relative mRNA expression of pro-inflammatory cytokines (TNFα, IL-1β, and IL-6) (Figure 7A–C) and upregulated the relative mRNA of anti-inflammatory cytokine IL-10 and Arg-1 (Figure 7D,E). Rg3 markedly decreased the relative expression of IL-1β and IL-6 (Figure 7B,C) and increased the expression of IL-10 (Figure 7D), while Rf significantly decreased the expression of IL-1β (Figure 7B). In the spleen, all three ginsenosides significantly decreased the level of IL-1β and increased the level of arginase1 (Arg-1) (Figure S1B,E). Rf and Rg3 decreased the relative expression of TNF-α and IL-6 (Figure S1A,C), and Rg1 and Rg3 significantly upregulated the expression of IL-10 (Figure S1D). Collectively, among these three ginsenosides, Rg1 was the most prominent in reducing colon inflammation.

To investigate whether the optimal effect of Rg1 on colitis was associated with macrophages, changes in macrophage polarization were further observed by flow cytometry (Figure 7F). Rg1 obviously decreased CD86^+^M1 macrophages (Figure 7J,H) and promoted the levels of CD206^+^M2 macrophages in DSS-induced mice (Figure 7I,J). These data indicated that Rg1 attenuated colitis, probably by reducing the polarization of M1 macrophages and inducing the polarization of M2 macrophages.

### 3.8. Regulation of Gut Metabolite Composition by Rg1

**Figure 8:**
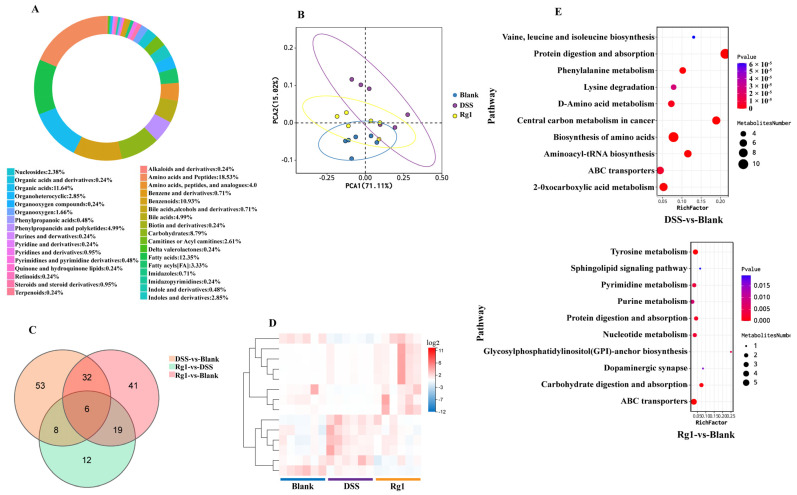
Rg1 supplementation recovered the composition of intestinal metabolites in DSS-induced mice. (**A**) Metabolites donut chart. Different colors represent different metabolite classifications. Percentages represent the percentage of metabolites belonging to a particular classification across all metabolites with classification. (**B**) PCA score graph. PC1 represents the first principal component and PC2 represents the second principal component. (**C**) Venn diagram of the differential metabolites. (**D**) Clustering on differential metabolites. The color represents the expression level, and blue to red corresponds to the expression level from low to high. (**E**) Metabolic pathway enrichment analysis bubble chart (*n* = 6). The X-axis RichFactor is the number of differential metabolites annotated in this pathway divided by all identified metabolites annotated in this pathway. The dot size represents the number of differential metabolites.

To further explore the mechanism of Rg1 attenuating DSS-induced colitis in mice, changes in 700 intestinal metabolites were analyzed. Seven hundred targeted metabolites, including amino acids and peptides, bile acids, fatty acids, carbohydrates, etc., were quantified by LC-MS, and 440 metabolites were identified in this study (Figure 8A). The principal component analysis (PCA) analysis results showed that the gut metabolite composition of DSS mice treated with Rg1 was more similar to that of the blank group (Figure 8B). Venn diagram results showed that 99 metabolites were radically changed after DSS induction, while 55 metabolites were changed after Rg1 treatment in DSS-induced mice, fourteen of these were common differential metabolites (Figure 8C). Cluster analysis of these 14 common differential metabolites showed that most of them were restored by Rg1 after being altered by DSS (Figure 8D). These differential metabolites by KEGG enrichment analysis showed that the main metabolic pathway changed by DSS was protein digestion and absorption and central carbon metabolism in cancer, while the pathway enriched by Rg1-regulated differential metabolites was glycosylphosphatidylinositol (GPI)-anchor biosynthesis, and their common metabolic pathway was ABC transporters (Figure 8E). Overall, these results suggested that Rg1 assumed a role in alleviating colitis by reversing DSS-altered metabolites, even though these shared metabolites are relatively rare.

### 3.9. Changes in Key Metabolites by Rg1

**Figure 9:**
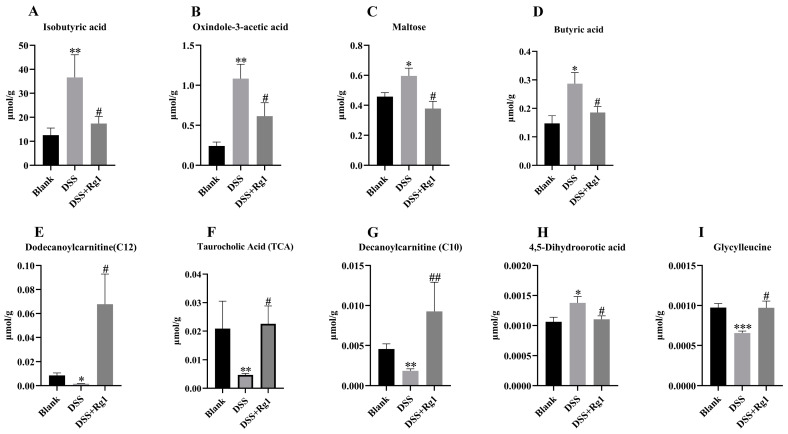
Rg1 treatment significantly altered key metabolites in mice with colitis. Changes in the level of (**A**) isobutyric acid, (**B**) oxindole-3-acetic acid, (**C**) maltose, (**D**) butyric acid, (**E**) dodecanoylcarnitine, (**F**) taurocholic acid, (**G**) decanoylcarnitine, (**H**) 4,5-dihydroorotic acid, and (**I**) glycylleucine were detected by LC-MS/MS after Rg1 treatment (*n* = 4). * *p* < 0.05, ** *p* < 0.01, and *** *p* < 0.001 compared with the blank group; ^#^ *p* < 0.05 and ^##^ *p* < 0.01 compared with the DSS group.

To further investigate the role of these metabolites in Rg1 remission of colitis, fourteen common metabolites were further analyzed. The results showed that nine key metabolites were significantly restored by Rg1 (Figure 9). The levels of isobutyric acid (Figure 9A), indole-3-acetic acid (Figure 9B), maltose (Figure 9C), butyric acid (Figure 9D), and 4,5-dihydroorotic acid (Figure 9H) in the intestine were significantly reduced by Rg1, while dodecanoylcarnitine (Figure 9E), taurocholic acid (Figure 9F), decanoylcarnitine (Figure 9G), and glycylleucine (Figure 9I) was significantly enriched. Therefore, these nine metabolites may be critical for Rg1 to reverse colitis.

## 4. Discussion

Modern pharmacological research shows that it has extraordinary effects on the treatment of many diseases, for example, cardiovascular diseases, gastrointestinal diseases, etc. [21,22]. Ginsenosides are the vital bioactive compounds in ginseng and have been studied for their outstanding anti-inflammatory effects [11]. In our study, the anti-inflammatory effects of six commonly studied ginsenosides, including Rg1, Rg3, Rf, Rd, Re, and Rb1, were also compared. These six ginsenosides were also present in the extract of Shen Ling Bai Zhu San n-butanol, which turned out to alleviate colitis in our previous study [3]. Our findings revealed which ginsenoside is responsible in ginseng and provided further evidence that ginsenoside attenuates colitis by regulating macrophage polarization and intestinal metabolism.

Rg1, Rg3, Rf, Rd, Re, and Rb1 have all been shown to alleviate colitis, but it is unclear which ginsenoside has the most prominent anti-inflammatory effect [23,24,25,26,27,28]. Firstly, the anti-inflammatory effect of ginsenoside was evaluated in the LPS-induced RAW264.7 cells model. In this study, our results indicated that Rg3 and Rf performed the best in reducing the expression of inflammatory cytokines, while Rg1, Rg3, and Rf exhibited the most significant inhibition in cell pyroptosis. Pyroptosis is initiated by caspase cleavage of GSDMD, and pyroptosis includes both classical and non-classical pathways, and the non-classical pathway is triggered by Caspase 11 [29]. Therefore, ginsenosides can reduce inflammation by inhibiting pyroptosis through non-classical pathways. Our previous study showed that Shen Ling Bai Zhu San alleviated colitis was significantly related to its inhibition of pyroptosis, which was consistent with the effect of ginsenosides on pyroptosis in the present study [29]. LPS stimulates macrophages to promote the production of ROS and then participates in many inflammatory processes, including mediating related cell signal transduction and activation of transcription factors, thereby regulating the occurrence and development of inflammatory responses [30]. ROS also induced the polarization of macrophages to the M1 type, and excessive lipid ROS is one of the necessary conditions for triggering ferroptosis [17,31]. In our study, Rg1 showed the most significant reduction in ROS production. Other ginsenosides also reduced inflammation to varying degrees. Additionally, Rg1, Rg3, and Rf also significantly inhibited the occurrence of ferroptosis. These results are in agreement with previous studies on the anti-inflammatory effects of ginsenosides [32]. However, in cell experiments, the anti-inflammatory effects of ginsenosides did not show a clear dose-dependent pattern, which may be related to the concentration we selected to screen for the anti-inflammatory effects of different ginsenosides at the same dose. Surprisingly, except for GPX4 expression, the dose-dependent effect of Rg1 was most pronounced among these ginsenosides. Meanwhile, through cell experiments, we also found that high doses of ginsenoside could activate the expression of related factors and lead to inflammation. The result of the current study also found that Rd produced a significant inhibition of cell viability at the concentration of 100 μM. Our results are consistent with studies indicating that ginseng or its active ingredients have been proven to be safe by various in vivo and in vitro tests and human clinical trials in recent decades, but can also produce significant toxicity when abused or used at increased doses [33,34]. Therefore, based on cellular results, Rg1, Rg3, and Rf were preeminent in anti-inflammatory effects in cells.

The body weight of mice, DAI, the length of the colon, colonic inflammation, and splenomegaly were the key indicators to measure DSS-induced colitis. In the present study, compared with Rg3 and Rf, Rg1 showed the most prominent effects in reversing body weight, reducing DAI, shortening colon and splenomegaly, and reducing colonic inflammatory cell infiltration in mice with colitis. Among them, the body weight after Rg1 treatment has not yet recovered to the level of the blank group, but it was consistent with previous studies [35]. Unexpectedly, only Rg1 significantly decreased the expression of pro-inflammatory cytokines and induced the expression of anti-inflammatory cytokines in colonic tissue. Rg1 also significantly decreased the concentration of pro-inflammatory cytokines in serum. These results are consistent with studies indicating that Rg1 significantly alleviates DSS-induced colitis [23,36]. The intestinal barrier is an important defense line against the invasion of intestinal pathogenic microorganisms, and tight junctions are an important part of the intestinal mechanical barrier. In the present study, Rg1 also rescued the DSS-induced intestinal barrier injury by upregulating the expression of claudin1, occludin, and ZO-1. Although no study has shown that Rg1 is the most effective among the six ginsenosides in alleviating DSS-induced colitis, Rg1 has the highest number of search results based on the keywords ginsenosides and colitis in the Web of Science. Macrophages in the intestinal lamina propria are mainly M1 type, which secrete pro-inflammatory cytokines, destroy the intestinal barrier function, and induce excessive inflammation [37]. However, M2 macrophages are a hostile force that can eliminate inflammation and promote tissue renewal [14]. Numerous previous studies have shown that inducing macrophage polarization from M1 to M2 alleviates the symptoms of colitis [38,39]. In this study, Rg1 significantly reduced the levels of M1 macrophages and activated polarization of M2 macrophages. In the meantime, ginsenosides were not found to promote inflammation or have potential side effects in vivo, which may be explored by more studies. Consistent with this, ginsenoside Rg1 has not been reported to cause adverse effects in vivo at the same dose, but Rb1 and Re were found to be embryotoxic at the concentration of 50 μg/mL in a mouse embryo model [40]. However, when the dose of ginsenoside increases to a certain extent, it will bring obvious cytotoxicity [41]. Taken together, Rg1 is expected to be a potential candidate for the treatment of colitis.

The gut microbiota is an influential player in the regulation of the gut. Its metabolites directly participate in a variety of biological processes, for example, short-chain fatty acids, tryptophan, etc. [42,43]. In this study, 700 targeted metabolites in the intestine, important metabolites involved in intestinal function, were detected, and 440 were obtained. With respect to these 440 metabolites, the composition of DSS-induced mice administered Rg1 gavage and DSS untreated mice were more similar. More importantly, nine key metabolites were restored by Rg1 in DSS mice. Curiously, butyric acid, isobutyric acid, and taurocholic acid were altered by DSS in previous studies [44,45,46], but in our study, the changes were reversed. Rg1 reduced the intestinal contents of butyric acid and isobutyric acid, which had been previously shown to be beneficial for colitis recovery [46,47]. This may be because short-chain fatty acids are not the key gut metabolites involved in the Rg1 response to colitis. In the present study, oxindole-3-acetic acid, maltose, 4,5-dihydroorotic acid, dodecanoylcarnitine, decanoylcarnitine, and glycylleucine were significantly changed by Rg1, but there is little research on the relationship between these metabolites and colitis. Among these metabolites, dodecanoylcarnitine and decanoylcarnitine were the most abundant metabolites and the most significantly altered intestinal metabolites by Rg1. Therefore, these six metabolites, especially dodecanoylcarnitine and decanoylcarnitine, may be the key metabolites for Rg1 to alleviate colitis, which requires more in-depth investigation.

## 5. Conclusions

In conclusion, our results indicated that among the six ginsenosides, Rg1, Rg3, and Rf were the most helpful in reducing LPS-induced inflammation in RAW264.7 cells. In vivo studies showed that Rg1 was superior to Rg3 and Rf in attenuating DSS-induced colitis, and this effect was related to macrophage polarization and gut metabolites. Our findings provide a promising approach for the treatment of colitis among so many ginsenosides. However, the role of these six metabolites in the reduction of intestinal inflammation by Rg1 needs to be further verified.

## Figures and Tables

**Table 1:** The gene sequence for RT-qPCR.

Target Gene	Direction	Sequence (5′–3′)
*TNF-α*	Forward	CTCACACTCACAAACCACCAAG
	Reverse	CAATGACTCCAAAGTAGACCTGC
*IL-1β*	Forward	AGGCAGGCAGTATCACTCATTG
	Reverse	CGTCACACACCAGCAGGTTATC
*IL-6*	Forward	AGTTGCCTTCTTGGGACTGATG
	Reverse	CATTGGAAATTGGGG TAGGAAG
*iNOS*	Forward	CTGCCAGGGTCACAACTTTAC
	Reverse	CAGCTCAGTCCCTTCACCAA
*IL-10*	Forward	TGGACAACATACTGCTAACCG
	Reverse	GGATCATTTCCGATAAGGCT
*Arg-1*	Forward	CGGGAGGGTAACCATAAGCC
	Reverse	CTTGGGAGGAGAAGGCGTTT
*IL-18*	Forward	TCAGACAACTTTGGCCGACT
	Reverse	TCAGTCTGGTCTGGGGTTCA
*Caspase-11*	Forward	ACAAACACCCTGACAAACCAC
	Reverse	CACTGCGTTCAGCATTGTTAAA
*GSDMA*	Forward	CCTCCTGGAGAAAAGCCAGG
	Reverse	TCTTCGTGCATCTCCCCAAC
*GSDMD*	Forward	CCATCGGCCTTTGAGAAAGTG
	Reverse	ACACATGAATAACGGGGTTTCC
*GSDMC*	Forward	CCTGCTAAAAGGAAGGAGACAGT
	Reverse	TTCCAGGCAGAAGCTGCGTT
*Claudin1*	Forward	TGGCATGAAGTGCATGAGGT
	Reverse	TTGTTTTCCGGGGACAGGAG
*Occludin*	Forward	ACGGACCCTGACCACTATGA
	Reverse	TCAGCAGCAGCCATGTACTC
*ZO-1*	Forward	CACTTCCAAAGACAGCGGGT
	Reverse	CCCAGCGTCTCGTAGTTCA
*β-Actin*	Forward	TGCTGTCCCTGTATGCCTCTG
	Reverse	CTGTAGCCACGCTCGGTCA

## Data Availability

The original contributions presented in this study are included in the article and Supplementary Materials. Further inquiries can be directed to the corresponding authors.

## Supplementary Materials

The following supporting information can be downloaded at: https://www.mdpi.com/article/10.3390/antiox14030283/s1. Figure S1: Changes in cytokines related to macrophage polarization by Rg1, Rg3, and Rf in the spleen.
