# Supplementary material for: Evaluation of Anti-Inflammatory Effects of Six Ginsenosides and Rg1 Regulation of Macrophage Polarization and Metabolites to Alleviate Colitis
Source: Antioxidants (Basel). 2025 Feb 27;14(3):283. doi: 10.3390/antiox14030283 (PMC11939151; doi:10.3390/antiox14030283)
Supplement: Supplementary file 1 [file antioxidants-14-00283-s001.zip › antioxidants-3454361-supplementary.pdf]

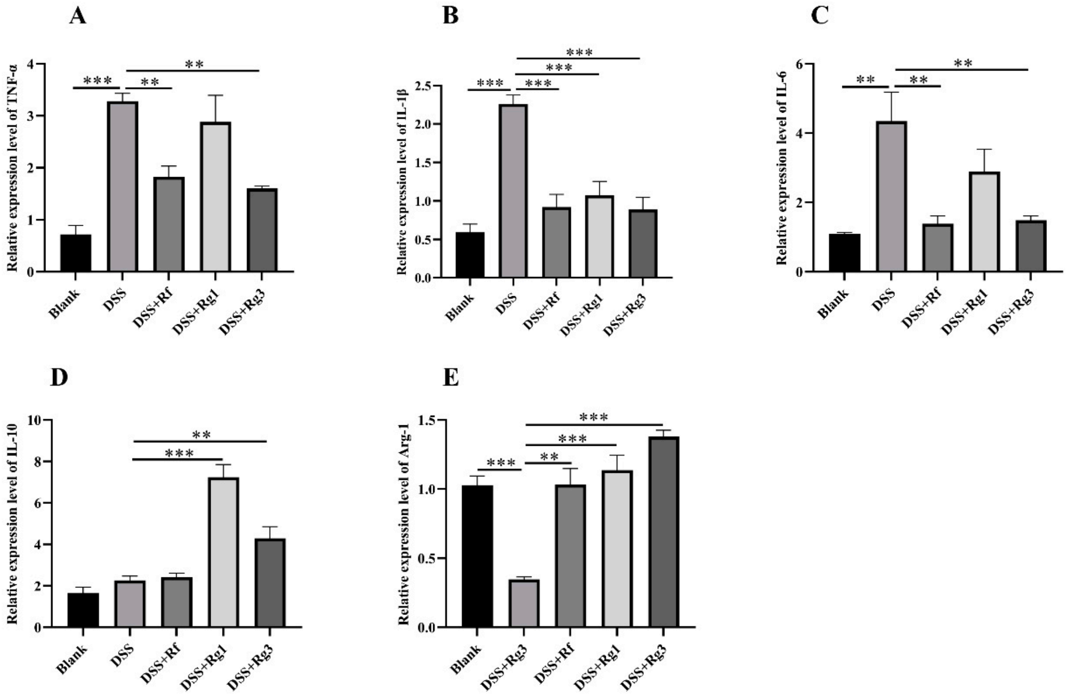


Figure S1. Changes in cytokines related to macrophage polarization by Rg1, Rg3 and Rf in spleen. The mRNA expression of (a) TNF-α, (b) IL-1β, (c) IL-6, (d) IL-10, and (e) Arg-1 in spleen tissue were determined by qPCR.
